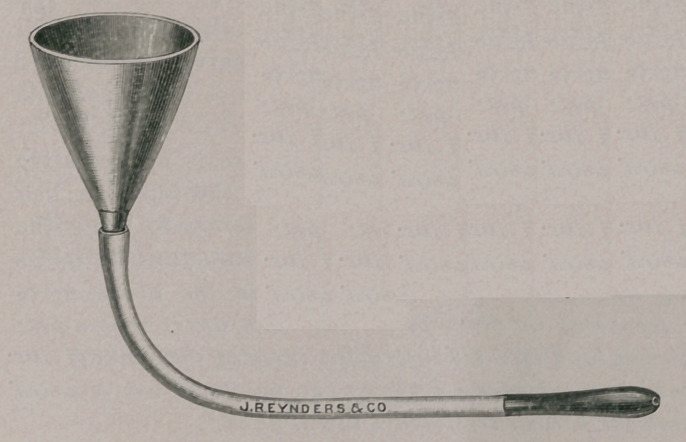# Cases

**Published:** 1891-11

**Authors:** Herbert Neher


### Cases.

### Foreign Body in Urethra.

By Herbert Neher, V. S.

My attention was called lately to a gray gelding 15½ hands, and very handsome. The symptoms presented were of a colicky nature; that is, he would roll, paw, look at his sides, etc., but the most noticable was his continual kicking, no increase of Temp. worth speaking about, he would stretch and try to urinate but could only produce a dribble. My diagnosis was obstruction "of some sort" in the bladder or urethra. I passed, or undertook to pass the catheter, but at a given point it would stop and I could not get it any farther. This point was just below the ischial arch. I then made an examination and found at this point a hard substance which I tried to work down, but could not get it to go either one way or the other. I recommended cutting down on it and removing it. I explained to the owner the consequences which *might* follow, and he consented. I injected a solution cocaine above and below the obstruction, cut down, and to my surprise, for I expected to see a urinary calculus, found it was the head of a catheter that had been passed by somebody and broken off, I think, in the bladder. Just when this was done, or by whom it was done, will always be a mystery, for the owner said he had owned the horse for almost a year. There was no particular deposit of the phosphates around it, but it was coated with a thick layer of mucus. My conclusion is that whoever passed the catheter used an old brittle one, and that in turning the ischial arch, it broke off and was pushed in the bladder.

Hence the necessity of using only instruments that are in first class order, and of the best make. I will add that the animal did very nicely, and made a complete recovery.

### Intestinal Calculus.

By Same.

I was called to treat a very large Blood Hound, that had been trying to defecate for one and a half days. He would suddenly start and run a short distance, squat, and try to have a passage; then he would set up a most unearthly howl; then he would be quiet for awhile, and go through the same performance again. His temperature was 104° F., his nose was dry and hot, membranes red and injected. I came to the conclusion that it was a case of constipation, and gave him a large dose of castor oil and told the owner that I would call the next day, and that in the meantime if the dog had a passage I wanted to see it. I called the next day and found the dog very uneasy, keeping up his occasional howling. I noticed that he would bite at the anus very often, so I got his master to hold him while I examined him there. I oiled the parts, my hand also, I then formed my hand cone-shaped and dilated the anus, and as I began to go further in I felt something hard. I happened to have a pig forceps with me and used it as a dilator. I then could look in the rectum and saw what I felt. I took a pair of dressing forceps, introduced them in the rectum, took hold of the object and pulled it out, which proved to be an intestinal calculus about 4 inches long, and very sharp at one end. It was made up principally of vegetable matter. The dog was in the habit of visiting the horse stable and eating horse manure, as I have often seen him do. He recovered his usual spirits in a few days and seems to be all right now.

---

### RECTAL INJECTION.

By Same.

I make it a point to inject by rectum every case of colic. I use such an instrument, as shown in the engraving. I like it much better than a syringe as it can be used quicker; the rubber hose is about 4 feet long, with a funnel attached to one end and a nozzle on the other. I have used nothing else for that purpose in almost four years, and would recommend it to the profession as a very satisfactory instrument.

## Figures and Tables

**Figure f1:**